# Reduced Graphene Oxide Coating LiFePO_4_ Composite Cathodes for Advanced Lithium-Ion Battery Applications

**DOI:** 10.3390/ijms242417549

**Published:** 2023-12-16

**Authors:** Qingao Zhang, Yu Zhou, Yulong Tong, Yuting Chi, Ruhua Liu, Changkai Dai, Zhanqing Li, Zhenli Cui, Yaohua Liang, Yanli Tan

**Affiliations:** 1 School of Chemical Science and Engineering, Qingdao University, Qingdao 266071, China; 2 Department of Agricultural and Biosystems Engineering, South Dakota State University, Brookings, SD 57007, USA

**Keywords:** LiFePO_4_, reduced graphene oxide coating, cathode materials, lithium-ion batteries

## Abstract

Recently, the application of LiFePO_4_ (LFP) batteries in electric vehicles has attracted extensive attention from researchers. This work presents a composite of LFP particles trapped in reduced graphene oxide (rGO) nanosheets obtained through the high-temperature reduction strategy. The obtained LiFePO_4_/rGO composites indicate spherical morphology and uniform particles. As to the structure mode of the composite, LFP distributes in the interlayer structure of rGO, and the rGO evenly covers the surface of the particles. The LFP/rGO cathodes demonstrate a reversible specific capacity of 165 mA h g^−1^ and high coulombic efficiency at 0.2 C, excellent rate capacity (up to 10 C), outstanding long-term cycling stability (98%) after 1000 cycles at 5 C. The combined high electron conductivity of the layered rGO coating and uniform LFP particles contribute to the remarkable electrochemical performance of the LFP/rGO composite. The unique LFP/rGO cathode provides a potential application in high-power lithium-ion batteries.

## 1. Introduction

In the context of global carbon neutrality, lithium-ion batteries (LIBs) have attracted massive attention around the world, especially in the fields of electric vehicles, mobile electronic devices, and biomedical devices [1]. It has been proved that the electrochemical properties of LIBs are mainly affected by cathode materials. Therefore, the exploration of cathode materials with high charge–discharge capacity and long-cycle stability is essential for the development of LIBs [2]. As a prevailing candidate for cathode material, LFPprovides the benefits of excellent thermal stability, relatively low cost, and environmental friendliness [3,4]. Nevertheless, the low electronic conductivity and lithium-ion diffusivity of LFP can restrict the energy density of LIBs [5]. So far, various strategies can be employed to overcome the above problems, such as particle size reduction [6,7], microstructure optimization [8,9], heteroatom doping [10,11], and integration with conductive carbon materials [4,12,13]. However, the first two solutions are at the expense of material flapping density and cumbersome procedures, which can lead to low energy density for scale-up applications [8]. Thus, the construction of carbonaceous LFP composites is the most efficient and practical strategy to deal with the above issues. Numerous carbon substrates were selected as conductive additives for LIBs, such as carbon black [14], metal–organic frameworks [8], graphene [15,16], biomass-derived carbon [17], activated carbon [18], and carbon nanotubes [19]. Particularly, two-dimensional graphene with a large specific surface area, outstanding mechanical strength, and unprecedented electrical conductivity have enabled it to function as a highly conductive matrix, which can provide effective electrical pathways for insulating LFP [16,20,21]. Furthermore, rGO derived from a graphene oxide precursor can provide a suitable substrate to immobilize LFP nanoparticles and prevent its agglomeration due to its flexible structure and large surface area, leading to effectively increased lithium ion and fast interfacial kinetics [22,23]. Meanwhile, rGO enhances the electrical conductivity of the electrode materials and reduces the transportation pathway between Li^+^ and electrons during repeated long cycling processes. Moreover, the two-dimensional carbon structure of rGO is much more suitable for transferring ballistic electrons even at ambient temperatures. In addition, rGO is preferred over other conductive additives since, as demonstrated in previous reports [24,25,26], the oxidized functionalities (C–OH, C=O, and epoxide groups) that decorate the rGO flakes provide grafting and seeding points for the nucleation of LFP particles [26,27]. More importantly, rGO can be prepared via different methods into free-standing films for binder-free and flexible conductive additives or electrodes of LIBs [28,29]. Nevertheless, conventional electrodes are usually assembled via coating with uniformly mixed slurries consisting of active materials, additives (carbon black or conductive graphite), and insulating binders (polytetrafluoroethylene (PTFE) or polyvinylidene difluoride (PVDF)) onto the metallic current collectors (Al foils, Cu foils, or nickel foam) [30]. First, those binders are expensive, volatile, and toxic, difficult to dispose of after use, and can result in safety issues during cycle processes [31]. In addition, binders and current collectors used are electrochemically inactive and increase the mass of the electrode. Thirdly, the binders impede the mobility of electrons and lithium ions, leading to the degradation of energy density, capacity, rate performance, and cycling stability [30]. On the contrary, free-standing electrodes can solve the complex and rigid fabrication process of traditional electrodes. Moreover, the binder-free and flexible structure has been proven to accelerate electrons/ions transport, maintain the mechanical properties and integrity of the electrode, and increase the energy density of the entire battery [31,32]. Therefore, the design and preparation of flexible and binder-free electrodes with enhanced performance have captured considerable attention. 

**Scheme 1:**
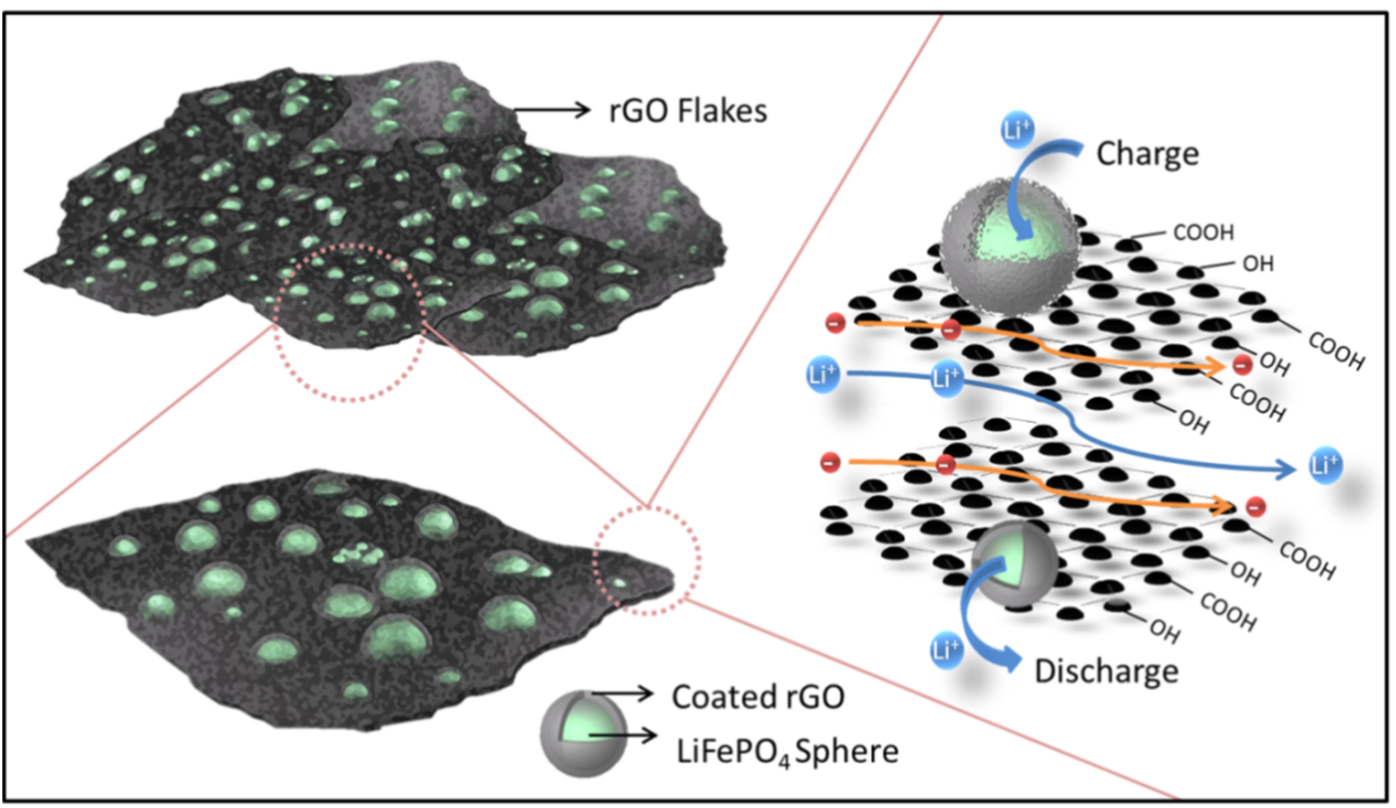
Schematic of the microstructure of LFP/rGO composites.

This work proposed the synthesis of free-standing reduced graphene oxide-coated LFP nanoparticles (denoted as LFP/rGO-X, X represents the calcination temperature). For this purpose, graphene oxide (GO) was first synthesized by utilizing Hummer’s method with slight modification and then obtained free-standing LFP/rGO-X composites by different high-temperature reductions after compounding with LFP. As shown in Scheme 1, the resulting composite possessed closely packed LFP nanoparticles individually embedded between rGO sheets. The LFP nanoparticles coated with thick rGO layers are homogeneously embedded in the rGO network. The rGO layers can provide a fast Li ion translation path, ensure high electron conductivity, and efficiently buffer volume change due to charge–discharge processes. Moreover, the conductive layered rGO sheet with controllable morphology can enhance the rapid transport of electrons and electrolyte ions, and the LFP nanoparticles on the surface of the composite can fully contact the electrolyte to carry out redox reactions, thereby achieving high capacity and long cycle stability. More importantly, free-standing LFP/rGO-X composites used as cathode materials for lithium-ion batteries without the addition of conductive agents and binders can reduce the cost of industrial production. When a free-standing LFP/rGO-300 composite was used as a binder-free and flexible cathode of LIBs, the capacity can reach 168 mA h g^−1^ at the current density of 0.1 C, and the capacity can still maintain 80 mA h g^−1^ after 1000 cycles at the 10 C current density, and the Coulombic efficiency remains above 98%.

## 2. Results and Discussion

**Figure 1:**
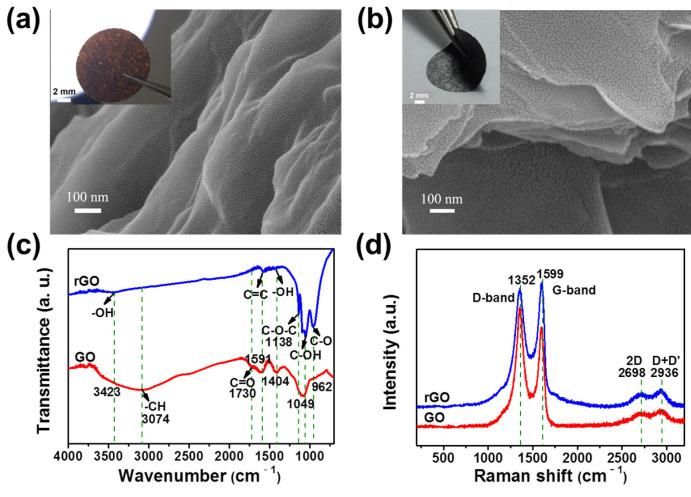
SEM images (the inset is a digital camera photo) of graphene oxide (**a**) and reduced graphene oxide (**b**); FTIR spectrum (**c**) and Raman spectrum (**d**) of graphene oxide and reduced graphene oxide.

The scanning electron microscope (SEM) was used to analyze the microstructures and morphologies of the GO and rGO samples. As shown in the inset of Figure 1a, the self-standing original GO film appears to be a brown-yellow color with some transparency and flexibility. An edge-side view SEM image of a representative GO sample is shown in Figure 1a, revealing the layered platelets comprised of curled nanosheets. GO is a random distribution of oxidized regions containing non-oxidized regions and oxygen-containing functional groups where many carbon atoms retain sp^2^ hybridization. Carrier concentration and mobility are both reduced because of functionalization since it disrupts the localized p-electrons and conjugated structure [33]. Therefore, GO reduction is concerned not only with removing oxygen-containing groups and other atomic-scale lattice defects but also with recovering the graphitic lattice’s conjugated network. When compared to the GO sample, the rGO exhibits a gleaming metallic shine due to an increase in electrical conductivity and visible light reflectance (Figure 1b inset) [34]. Meanwhile, the rGO film still maintained strength and flexibility. The SEM image in Figure 1b shows rGO nanosheets separated from each other, and the layered structure with some intervals becomes more obvious. Some of the rGO nanosheets even exfoliated from the main body to form small rGO slices. This unique structure is beneficial for ion and electron conductivity.

The Fourier transform infrared (FTIR) spectrum (Figure 1c) was utilized to recognize the functional groups of GO and rGO samples. The peaks of GO are composed of oxygenic groups that are covalently attached, including -OH (3423 cm^−1^, 1404 cm^−1^) [35], C=O (1730 cm^−1^) [36], C-OH (1049 cm^−1^) [37]. Upon comparison with GO, the peaks of -OH, C=O, and C–OH in the rGO sample weakened and even disappeared, illustrating that GO can be reduced after the calcination at 300 ℃. Meanwhile, the intensity of C–O–C (1138 cm^−1^) [38] and C–O (962 cm^−1^) [36] increased.

The GO and rGO were detected using Raman spectra (Figure 1d). Single-layer graphene’s Raman spectra displayed two distinctive peaks. The E_2g_ vibrational mode of sp^2^-bonded carbon is responsible for the G-peak at 1580 cm^−1^. The phonon scattering at the zone boundary is responsible for the second-order vibration that produces the two-dimensional (2D) peak at 2670 cm^−1^. For rGO, a frequency redshift of 20–30 cm^−1^ is related to a broadening of the 2D peak (2698 cm^−1^), and this is caused by electronic interactions between the layers [39]. The defective graphitic and disordered carbon structures are associated with the peak at about 1352 cm^−1^ (D band), which is attributed to the breathing mode of κ-point phonons with A_1g_ symmetry. The I_D_/I_G_ ratios of the intensity of two peaks partially depend on the graphitization degrees. The I_D_/I_G_ value of the rGO material is 0.95, which is much lower than 1.20 for GO material, further proving the sp^2^ carbon increase of rGO membranes [34]. The observation of the D + D’ (2936 cm^−1^) band in both GO and rGO materials revealed their defective structure [40].

**Figure 2:**
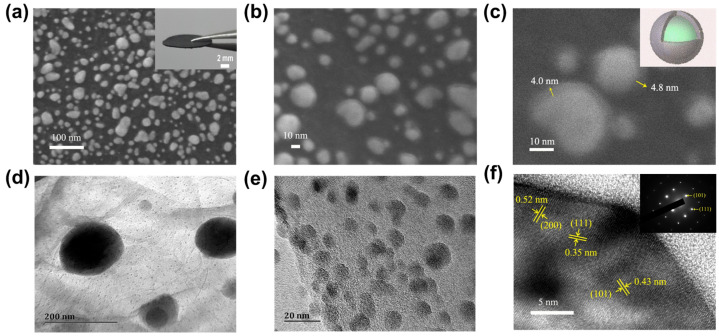
SEM images (**a**) (the inset is a photo), (**b**,**c**) (the inset is a simulation model), TEM images (**d**,**e**), and HRTEM images (**f**) (the inset is the selected area electron diffraction pattern) of LFP/rGO-300 composite.

Four typical kinds of LFP/rGO-X composites, LFP/rGO-200, LFP/rGO-300, LFP/rGO-400, and LFP/rGO-500, were investigated corresponding to the different calcination temperatures of 200, 300, 400, and 500 °C for 1 h (detail is provided in Section 3). As shown in the optical photo of the LFP/rGO-300 composite (Figure 2a inset), the LFP/rGO-300 is free-standing, and the battery can be assembled without a current collector. The LFP nanoparticles with uniform size can reduce the diffusion pathway of Li^+^ and prevent the restacking of rGO sheets, which accelerates Li^+^ transmission and improves the electrochemical performance of LFP nanoparticles (Figure 2a,b). As shown in Figure 2c, the continuous amorphous rGO layer is approximately 4–5 nm thick and uniformly covers the surface of LFP, forming a typical core-shell structure (Figure 2c inset). The LFP particles are homogeneously embedded in the rGO network, and the rGO networks interconnect each other to form a bigger network (Figure S1a,b). The electronic conductivity is mostly determined by the graphitic lattice’s long-range conjugated network. Hence, this unique structure would ensure a good connection between the particles, thereby increasing electronic conductivity [41]. The as-obtained micrographs of LFP/rGO-200, LFP/rGO-400, and LFP/rGO-500 composites are depicted in Figure S2. The LFP nanoparticles of all samples are uniform, and the particle size increases with increasing calcination temperature. Therefore, the morphologies and the particle sizes of the obtained LFP/rGO composite can be controlled by modifying the calcination temperature so as to lead to different electrochemical performances.

**Figure 3:**
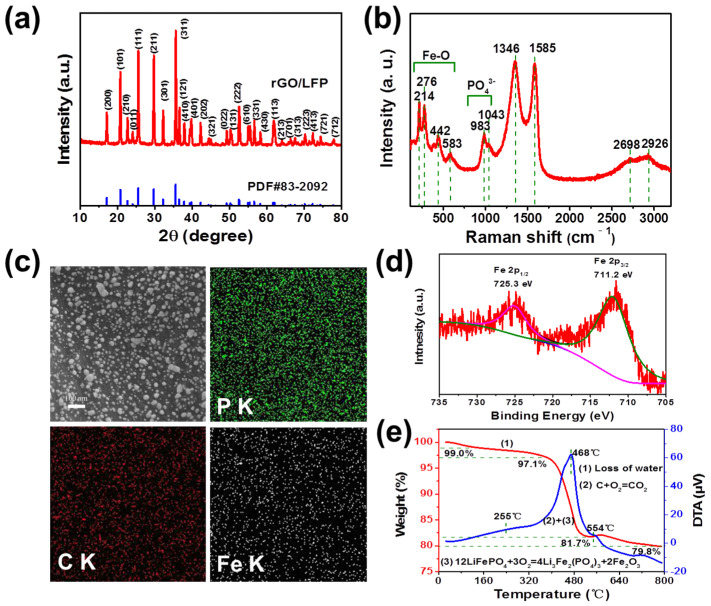
XRD pattern (**a**), Raman spectrum (**b**), EDS element mapping (Fe, P, C) (**c**), XPS spectra ofFe 2p (**d**), and TGA curves (**e**) for LFP/rGO-300 composite.

The morphology structures and phase analyses of LFP/rGO-300 were identified by the transmission electron microscope (TEM) and high-resolution TEM (HRTEM). Obviously, the shape of the LFP nanoparticle is spherical, and the size is about 20–30 nanometers (Figure 2d). As shown in Figure 2e, a coating layer is covered on the surface of the LFP nanoparticle. It can be clearly seen that the crystal lattice fringe spacing of 0.35, 0.43, and 0.52 nm represent the crystal planes of (111), (101), and (200) of the orthorhombic system LFP (Figure 2f) [42,43,44], respectively, which coincides with those obtained using X-ray diffractometry (XRD) (Figure 3a). The HRTEM images and corresponding selected area electron diffraction (SEAD) pattern (Figure 2f inset) of the LFP nanoparticles reveal the formation of a single-crystalline LFP phase inside the LFP/rGO-300 composite [45].

Figure 3a shows the XRD pattern of the LFP/rGO-300 composite; the main diffraction peaks can be indexed to the orthorhombic LFP phase (JCPDS No. 83-2092) [44]. The shape of the diffraction peak is sharp and prominent, denoting a crystallization structure of LFP [43]. These results are consistent with the HRTEM and SEAD (Figure 2f). The XRD pattern also shows no crystalline graphite phase, which is indicative of the amorphous nature of the rGO matrices used in the composite [46].

The structure of the LFP/rGO-300 composite was further measured by Raman spectroscopy (Figure 3b). The peaks at 100–600 cm^−1^ represent the Raman vibrations of Fe-O [46]. The peaks in the wavenumber range of 900–1100 cm^−1^ can be ascribed to the intramolecular stretching modes of the PO_4_^3−^ [43]. The value of the I_D_/I_G_ ratio is 1.01, which is near that of pure rGO (0.95). It reveals that the intervention of LFP to rGO material only slightly affected the ordered graphitic structure. The elemental mapping clearly reveals a homogeneous distribution of the P, C, and Fe (Figure 3c). The superior electrochemical performance of LFPs can be attributed to their uniformly distributed nanoparticles and suitable carbon coating layers [47].

**Figure 4:**
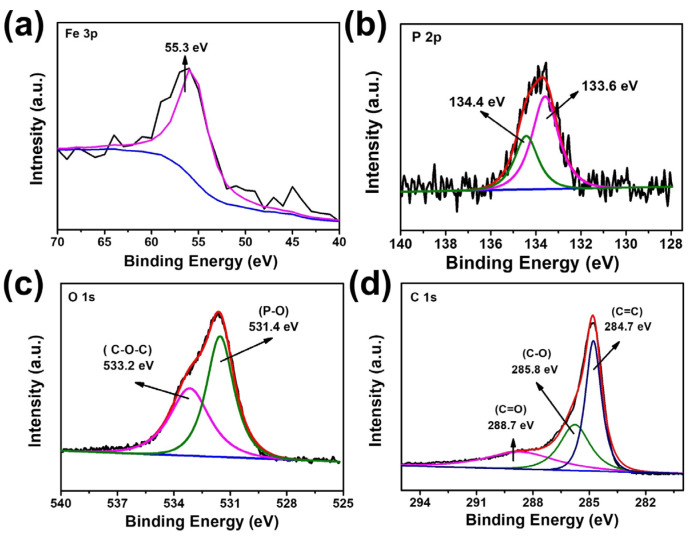
XPS spectra of Fe 3p (**a**), P 2p (**b**), O1s (**c**), and C1s (**d**) for the LFP/rGO-300 composite.

The X-ray photoelectron spectroscopy (XPS) was utilized to determine the chemical states on the LFP/rGO-300 surface. As shown in Figure 3d and Figure 4a, the specific shape of Fe 2p (725.3 eV and 711.2 eV) together with the peak of Fe 3p (55.3 eV) can determine the oxidation state of Fe as +2 [44,48]. The P 2p spectrum (Figure 4b) can be decomposed into two spin-orbit components at 133.6 eV and 134.4 eV. Meanwhile, these positions of the P 2p spectrum, together with the peak of O 1s at 531.4 eV (Figure 4c), are in good agreement with the presence of (PO_4_)^3−^ groups [49]. The C 1s spectra (Figure 4d) can be divided into three peaks: C=O (288.7 eV), C-O (285.8 eV), and C=C (284.7 eV), proving the connection mode of C and other functional groups [45].

Figure 3e presents thermogravimetric analyses (TGA) and differential thermal analysis (DTA) profiles of LFP/rGO-300. Obviously, three stages of weight loss were observed. The initial weight loss (1.4 wt%) before 200 °C is attributed to the evaporation of crystal and adsorbed water. The DTA curve revealed a broad exothermic peak in the range of 200 to 650 °C [50], associated with the 17.9 wt% of primary weight loss, corresponding to the joint results of the combustion of carbon to CO_2_ and the oxidation of LFP into Li_3_Fe_2_(PO_4_)_3_ and Fe_2_O_3_ [42]. At about 650 °C, the LFP/rGO-300 sample finished the oxidizing reactions in air, and the mass of the sample was nearly constant. The final weight loss in the TGA is about 20.2 wt%. According to theoretical calculations and literature reports, there is a weight increase of about 5.1 wt% when heating LFP up to 600 °C in air [42,51]. Therefore, the rGO weight percentage in the LFP/rGO-300 composite could be estimated as 23.9 wt% (20.2 wt% + 5.1 wt% − 1.4 wt%). 

**Figure 5:**
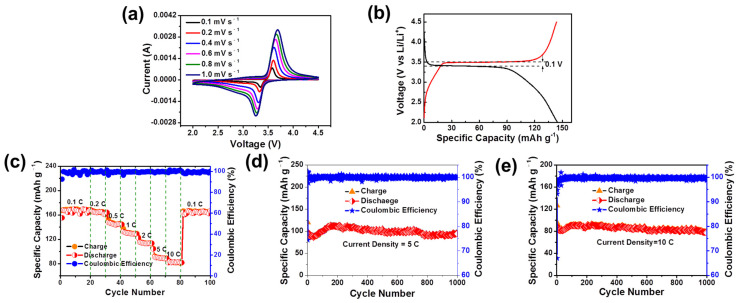
Electrochemical properties of the LFP/rGO-300 cathodes for LIBs. (**a**) Cyclic voltammetry curves at various scan rates of 0.1, 0.2, 0.4, 0.6, 0.8, and 1.0 mV s^−1^, (**b**) Galvanostatic charge–discharge profiles for the first cycle at 1 C. (**c**). Rate performances at different current densities from 0.1 to 10 C. (**d**) The charge–discharge cycling performances at 5 C. (**e**) The charge–discharge cycling performances at 10 C.

To detect the potential of the LFP/rGO composite as the cathode for LIBs, the electrochemical property of the LFP/rGO-300 was explored in coin-type cells between 2.0 and 4.5 V vs. Li/Li^+^. Cyclic voltammograms (CV) of LFP/rGO-300 were recorded at different scan rates, as shown in Figure 5a. It shows one distinct anodic (3.6 V) peak and one cathodic (3.4 V) peak, which is assigned to the Fe^2+/3+^ redox couple [52]. Figure 5b shows the charge–discharge curves of the LFP/rGO-300 composite at 1 C (1C = 170 mA g^−1^). The calculated specific capacities were based on the mass of LFP. The curves exhibit a typical plateau at approximately 3.4 V and a small voltage difference (0.1 V) between the plateaus of the charge–discharge potential, indicating poor polarization, excellent reversibility, and superior electrochemical kinetics [53]. For comparison, the charge–discharge measurements of the LFP/rGO-200, LFP/rGO-400, and LFP/rGO-500 electrodes were also carried out at the same scan rate (Figure S3a). It was found that LFP/rGO-300 exhibited the 144 mA h g^−1^ of the highest discharge capacity and a flat discharge profile of around 3.4 V in all these materials.

**Figure 6:**
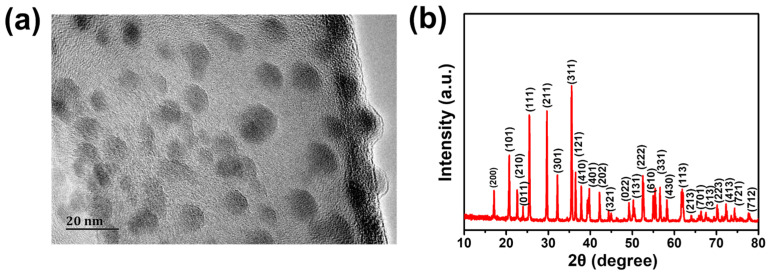
TEM image (**a**) and XRD pattern (**b**) of the LFP/rGO-300 electrode after 1000 cycles at a current density of 5 C.

At various current densities, the LFP/rGO-300 electrode rate performance is depicted in Figure 5c. The LFP/rGO-300 electrode exhibits the specific capacity of 168, 165, 148, 133, 116, and 93 mA h g^−1^, respectively, at 0.1, 0.2, 0.5, 1, 2, and 5 C. When the charge–discharge rate increases to 10 C, the high reversible capacity reaches up to 83 mA h g^−1^. When the charge–discharge rate comes back to 0.1 C, the specific capacity can maintain 166 mA h g^−1^ once again. The charge–discharge efficiency of the whole process remains above 97%, proving the good rate performance of the composite. The specific capacity of the LFP/rGO-300 electrode experiences almost no fading after 1000 cycles at 5 C (Figure 5d). Moreover, the discharge-specific capacity of the electrode can remain 80 mA h g^−1^ after 1000 cycles at 10 C of high current rate with an efficiency of around 99% (Figure 5e). The specific capacity reduces by only 9%, showing excellent cycle stability. In addition, the TEM image (Figure 6a) and XRD pattern (Figure 6b) of the LFP/rGO-300 electrode after 1000 cycles at 5 C were tested. Obviously, no changes can be observed in the structure, size, morphology, and main diffraction peaks of the LFP/rGO-300 electrode, delivering the structural stability of the composite after the long cycling.

This is mainly due to the unique structure of LFP/rGO-300 composite materials. As shown in Table S1, the Li-ion storage of the LFP/rGO-300 composite is better than or comparable to other LFP-based electrodes in previous literature reports [8,10,12,13,17,19,54,55,56,57,58,59,60,61,62,63,64,65,66,67]. The layered rGO matrix and carbon coating ensure the high structural stability and electrical conductivity of the composite material. Therefore, the material could not reduce the lithium storage performance due to the structure of pulverization, drastic changes in volume, or the structural damage of LFP agglomeration during charge–discharge processes. 

For comparison, the cyclic stability and rate performance of the LFP/rGO-200, LFP/rGO-400, and LFP/rGO-500 electrodes were also carried out at the same measurement condition (Figure S3b,c). Obviously, the cyclic stability and rate performance of LFP/rGO-300 are superior to that of the other three materials. The size and uniform distribution of LFP particles can be beneficial to exert pseudo-capacitance and effectively prevent the agglomeration of LFP particles, leading to excellent electrochemical performance. The LFP particle size of LFP/rGO-400 and LFP/rGO-500 is too large, which could reduce the effective utilization of LFP and increase the quality of effective materials, thus affecting the overall discharge-specific capacity of the composite materials. Similarly, the LFP particle size of LFP/rGO-200 is too small, and too few LFP particles can play a role in the electrochemical performance, resulting in a lower composite material’s specific capacity. Therefore, the structure of the composite material, such as the mass ratio and particle size of LFP, can be controlled by synthetic methods to control the electrochemical performance of LFP/rGO composites.

Figure S3d presents the electrochemical impedance spectroscopy (EIS) data of LFP/rGO-200, LFP/rGO-300, LFP/rGO-400, and LFP/rGO-500 electrodes. In the region of medium–high frequency, the radius of the semicircle increases with increasing calcination temperature. This means that the charge transfer resistance (R_ct_) of these four samples becomes larger. As is well known, the inherent conductivity of LFP is relatively low. The higher calcination temperature can increase the diameter of LFP particles, thus leading to much higher internal electrode resistance. In the region of low frequency, the trend of the diagonal balance deviating from the imaginary axis is more obvious with the increase of the calcination temperature, indicating the worse the capacitor performance of these composites. However, the internal resistance of LFP/rGO-300 is close to that of LFP/rGO-200 (200 Ω), and the oblique line is most closely parallel to the imaginary axis. Therefore, the LFP/rGO-300 showed the best electrochemical performance of the four LFP/rGO composites. EIS data once again demonstrate that calcination temperature can effectively affect the structure and electrochemical properties of LFP/rGO electrodes.

## 3. Materials and Methods

### 3.1. Synthesis of Graphene Oxide (GO)

GO was prepared according to the Hummer’s method [68]. P_2_O_5_ (10 g), K_2_S_2_O_8_ (10 g), and 20 g of graphite flakes were added to the mixture of 60 mL concentrated H_2_SO_4_ under stirring at 80 °C for 6 h followed by cooling and diluting with deionized (D.I) water. Then, the mixture was filtered using vacuum filtration and dried at 25 °C. Six grams of GO precursor and 18 g of KMnO_4_ were added dropwise to 140 mL of H_2_SO_4_ with stirring for 2 h in an ice-water bath followed by heating at 40 °C and stirred for a further 2 h. D.I water (700 mL) and H_2_O_2_ (15 mL) were added into the mixture and kept 48 h to settle the GO. It was then washed several times and centrifuged with diluted HCl and D.I water. Finally, the obtained GO was dispersed in water (600 mL) to form a uniform gel.

### 3.2. Synthesis of LiFePO_4_/Reduced Graphene Oxide (LFP/rGO-X) Composites

Next, 0.05 M LiOH∙H_2_O, H_3_PO_4,_ and FeSO_4_∙7H_2_O solutions were added with vigorous stirring. After that, 100 mL of diluted H_2_O_2_ and 3M NH_3_∙H_2_O solution was slowly added into the mixture at pH = 2. After stirring for 2 h, the desired amount of GO was added and stirred for a further 2 h. The mass ratio of GO in the precursor was 20 wt%. Then, the solution was centrifuged for 4 h to undergo exfoliation via sonication. Aluminum foil was coated with the slurry and dried for 24 h at room temperature. After drying, the membrane was removed from the aluminum foil and cut into a round shape with a diameter of 1.1 cm. Finally, the round membrane was heated to 300 °C at a rate of 2 °C min^−1^ in a tube furnace and kept for 1 h under a flowing nitrogen atmosphere (named LFP/rGO-300). For comparison, the LFP/rGO-200, LFP/rGO-400, and LFP/rGO-500 samples were fabricated after the calcination at 200 °C, 400 °C and 500 °C for 1 h, respectively.

### 3.3. Materials Characterization

Microstructures were observed using SEM(Quanta-250 FEG, Hillsboro, OR, USA ) with a voltage of 5 kV and current of 10 μA and 8~10 mm of working distance. Observations of HRTEM were conducted at 200 kV using a JEM-2100F microscope (JEOL, Showima City, Tokyo, Japan) equipped with a field-emission gun. FTIR was acquired on a Nicolet 6700 (ThermoFisher Scientific, Waltham, MA, USA). The Raman spectrum was performed on a microscopic confocal Raman spectrometer (LabRAM HR800, HORIBA Jobin, Paris, France) under a backscattering geometry (λ = 514 nm). The crystalline structure and phase purity of the sample were determined using powder XRD (Labx XRD-6000, Shimadzu, Kyoto, Japan) with Cu Kα radiation (λ = 1.5418 Å) in the range of 10° ≤ 2θ ≤ 70° (step 0.02° and scan speed 3° min^−1^) at room temperature. -XPS- analysis was conducted using an Al Ka (150 W) monochromatic X-ray source (ESCALAB 250, ThermoFisher Scientific, Waltham, MA, USA). The thermal analysis was determined under air atmosphere by SDTQ600 (TA Instruments, New Castle, DE, USA) at a heating rate of 10 °C min−1^−1^ to 800 °C.

### 3.4. Electrochemical Measurements

The electrochemical measurement experiments were performed at room temperature on a CHI660D electrochemical workstation (Shanghai Chenhua Instruments, Co., Shanghai, China). To conduct the charge–discharge tests, CR2032 coin cells were used. A total of 2032 stainless steel coin cells with LFP/rGO electrodes and Li metal with a porous polymeric separator were assembled with 1.0 M LiPF_6_ dissolved in ethylene carbonate (EC)-dimethyl carbonate (DEC) (1:1 of volume ratio) organic electrolyte. The mass of active material loaded on the current collector was 2.0 mg. The working electrode was subjected to EIS investigations at a frequency range of 0.01 Hz and 100 Hz. 

## 4. Conclusions

In summary, we first synthesized GO via the modified Hummer’s method and then rationally designed layer rGO-coated LFP nanoparticles with the typical core-shell structure through the high-temperature reduction strategy. The obtained LFP/rGO-300 composite exhibited a nearly spherical shape with a uniform diameter. LFP particles were completely covered with an amorphous rGO layer approximately 4–5 nm thick. LFP/rGO-300 composite used as binder-free and flexible cathode materials for lithium-ion storage delivered 168 mA h g^−1^ of high specific capacity at 0.1 C, exceptional cyclic stability (98% capacity retention after 1000 cycles at 5 C), and excellent rate performance (83 mA h g^−1^ at 10 C). Therefore, the combination of uniform LFP nanoparticles and rGO coating of the flexible and free-standing LFP/rGO composites can improve the stability and conductivity of the composites, create an elastic retention space to adapt to increasing the volume expansion/contraction during charge–discharge processes and provide a strong guarantee for electrochemical stability, thereby achieving high capacity and long cycle stability. This work opens an exciting avenue for free-standing LFP-based cathode materials in the prospect of practical application for lithium-ion batteries.

## Data Availability

Data are contained within the article and Supplementary Materials.

## Supplementary Materials

The supporting information can be downloaded at: https://www.mdpi.com/article/10.3390/ijms242417549/s1.

## References

[1] Etacheri V., Marom R., Elazari R., Salitra G., Aurbach D. (2011). Challenges in the development of advanced Li-ion batteries: A review. Energy Environ. Sci..

[2] He W., Guo W., Wu H., Lin L., Liu Q., Han X., Xie Q., Liu P., Zheng H., Wang L. (2021). Challenges and Recent Advances in High Capacity Li-Rich Cathode Materials for High Energy Density Lithium-Ion Batteries. Adv. Mater..

[3] Li H., Peng L., Wu D., Wu J., Zhu Y.J., Hu X. (2019). Ultrahigh-capacity and fire-resistant LiFePO_4_-based composite cathodes for advanced lithium-ion batteries. Adv. Energy Mater..

[4] Guo L., Zhang Y., Wang J., Ma L., Ma S., Zhang Y., Wang E., Bi Y., Wang D., McKee W.C. (2015). Unlocking the energy capabilities of micron-sized LiFePO4. Nat. Commun..

[5] Dong G., Mao Y.-Q., Li Y.-Q., Huang P., Fu S.-Y. (2022). MXene-carbon nanotubes-cellulose-LiFePO_4_ based self-supporting cathode with ultrahigh-area-capacity for lithium-ion batteries. Electrochim. Acta.

[6] Chen S.-P., Lv D., Chen J., Zhang Y.-H., Shi F.-N. (2022). Review on defects and modification methods of LiFePO_4_ cathode material for lithium-ion batteries. Energy Fuels.

[7] Chen Y., Xiang K., Zhou W., Zhu Y., Bai N., Chen H. (2018). LiFePO_4_/C ultra-thin nano-flakes with ultra-high rate capability and ultra-long cycling life for lithium ion batteries. J. Alloys Compd..

[8] Lin J., Sun Y.-H., Lin X. (2021). Metal-organic framework-derived LiFePO_4_ cathode encapsulated in O, F-codoped carbon matrix towards superior lithium storage. Nano Energy.

[9] Wang J., Yang J., Tang Y., Liu J., Zhang Y., Liang G., Gauthier M., Chen-Wiegart Y.C.K., Banis M.N., Li X. (2014). Size-dependent surface phase change of lithium iron phosphate during carbon coating. Nat. Commun..

[10] Ma Z., Zuo Z., Li L., Li Y. (2022). Unleash the capacity potential of LiFePO_4_ through rocking-chair coordination chemistry. Adv. Funct. Mater..

[11] Okita N., Kisu K., Iwama E., Sakai Y., Lim Y., Takami Y., Sougrati M.T., Brousse T., Rozier P., Simon P. (2018). Stabilizing the structure of LiCoPO_4_ nanocrystals via addition of Fe^3+^: Formation of Fe^3+^ surface layer, creation of diffusion-enhancing vacancies, and enabling high-voltage battery operation. Chem. Mater..

[12] Shih J.-Y., Lin G.-Y., Li Y.-J.J., Hung T.-F., Jose R., Karuppiah C., Yang C.-C. (2022). Operando investigation on the fast two-phase transition kinetics of LiFePO_4_/C composite cathodes with carbon additives for lithium-ion batteries. Electrochim. Acta.

[13] Zhang B., Wang S., Liu L., Wang J., Liu W., Yang J. (2022). Facile synthesis of S-doped LiFePO_4_@N/S-doped carbon core-shell structured composites for lithium-ion batteries. Nanotechnology.

[14] Qi X., Blizanac B., DuPasquier A., Oljaca M., Li J., Winter M. (2013). Understanding the influence of conductive carbon additives surface area on the rate performance of LiFePO_4_ cathodes for lithium-ion batteries. Carbon.

[15] Song W., Liu J., You L., Wang S., Zhou Q., Gao Y., Yin R., Xu W., Guo Z. (2019). Re-synthesis of nano-structured LiFePO_4_/graphene composite derived from spent lithium-ion battery for booming electric vehicle application. J. Power Sources.

[16] Geng J., Zhang S., Hu X., Ling W., Peng X., Zhong S., Liang F., Zou Z. (2022). A review of graphene-decorated LiFePO_4_ cathode materials for lithium-ion batteries. Ionics.

[17] Sim G.S., Nanthagopal M., Santhoshkumar P., Park J.W., Ho C.W., Shaji N., Kim H.K., Lee C.W. (2022). Biomass-derived nitrogen-doped carbon on LiFePO_4_ material for energy storage applications. J. Alloys Compd..

[18] Tsuda T., Ando N., Utaka T., Kojima K., Nakamura S., Hayashi N., Soma N., Gunji T., Tanabe T., Ohsaka T. (2019). Improvement of high-rate performance of LiFePO_4_ cathode with through-holed LiFePO_4_/Activated carbon hybrid electrode structure fabricated with a pico-second pulsed laser. Electrochim. Acta.

[19] Liu Y., Zhang H., Huang Z., Wang Q., Guo M., Zhao M., Zhang D., Wang J., He P., Liu X. (2022). Understanding the influence of nanocarbon conducting modes on the rate performance of LiFePO_4_ cathodes in lithium-ion batteries. J. Alloys Compd..

[20] Novoselov K.S., Geim A.K., Morozov S.V., Jiang D., Zhang Y., Dubonos S.V., Grigorieva I.V., Firsov A.A. (2004). Electric field effect in atomically thin carbon films. Science.

[21] Ramanathan T., Abdala A.A., Stankovich S., Dikin D.A., Herrera-Alonso M., Piner R.D., Adamson D.H., Schniepp H.C., Chen X., Ruoff R.S. (2008). Functionalized graphene sheets for polymer nanocomposites. Nat. Nanotechnol..

[22] David L., Bhandavat R., Barrera U., Singh G. (2016). Silicon oxycarbide glass-graphene composite paper electrode for long-cycle lithium-ion batteries. Nat. Commun..

[23] Wu C., Kopold P., Aken P.A., Maier J., Yu Y. (2017). High performance graphene/Ni_2_P hybrid anodes for lithium and sodium storage through 3D yolk-shell-like nano-structural design. Adv. Mater..

[24] Wang B., Al Abdulla W., Wang D., Zhao X.S. (2015). Three-dimensional porous LiFePO_4_ cathode material modified with nitrogen-doped graphene aerogel for high-power lithium ion batteries. Energy Environ. Sci..

[25] Wang B., Liu A., Al Abdulla W., Wang D., Zhao X.S. (2015). Desired crystal oriented LiFePO_4_ nanoplatelets in situ anchored on a graphene cross-linked conductive network for fast lithium storage. Nanoscale.

[26] Longoni G., Panda J.K., Gagliani L., Brescia R., Manna L., Bonaccorso F., Pellegrini V. (2018). In situ LiFePO_4_ nano-particles grown on few-layer graphene flakes as high power cathode nanohybrids for lithium-ion batteries. Nano Energy.

[27] Kumar P.V., Bernardi M., Grossman J.C. (2013). The impact of functionalization on the stability, work function, and photoluminescence of reduced graphene oxide. ACS Nano.

[28] Liu H., Wang H., Zhang X. (2015). Facile fabrication of freestanding ultrathin reduced graphene oxide membranes for water purification. Adv. Mater..

[29] Dikin D.A., Stankovich S., Zimney E.J., Piner R.D., Dommett G.H.B., Evmenenko G., Nguyen S.T., Ruoff R.S. (2007). Preparation and characterization of graphene oxide paper. Nature.

[30] Liu W., An Y., Wang L., Hu T., Li C., Xu Y., Wang K., Sun X., Zhang H., Zhang X. (2023). Mechanically flexible reduced graphene oxide/carbon composite films for high-performance quasi-solid-state lithium-ion capacitors. J. Energy. Chem..

[31] He D., Zhang Y., Cao D., Sun M., Xia J., Yang Y., Ding Y., Chen H. (2022). A flexible free-standing FeF_3_/reduced graphene oxide film as cathode for advanced lithium-ion battery. J. Alloys Compd..

[32] Yuan J., Zhu J., Wang R., Deng Y., Zhang S., Yao C., Li Y., Li X., Xu C. (2020). 3D few-layered MoS_2_/graphene hybrid aerogels on carbon fiber papers: A free-standing electrode for high-performance lithium/sodium-ion batteries. Chem. Eng. J..

[33] Pei S., Cheng H. (2012). The reduction of graphene oxide. Carbon.

[34] Pei S., Zhao J., Du J., Ren W., Cheng H. (2010). Direct reduction of graphene oxide films into highly conductive and flexible graphene films by hydrohalic acids. Carbon.

[35] Wu X., Wen T., Guo H., Yang S., Wang X., Xu A. (2013). Biomass-derived sponge-like carbonaceous hydrogels and aerogels for supercapacitors. ACS Nano.

[36] Grzyb B., Hildenbrand C., Berthon-Fabry S., Begin D., Job N., Rigacci A., Achard P. (2010). Functionalisation and chemical characterization of cellulose-derived carbon aerogels. Carbon.

[37] Sun X., Li Y. (2004). Colloidal carbon spheres and their core/shell structures with noble-metal nanoparticles. Angew. Chem. Int. Ed..

[38] Yang C., Gao Q., Tian W., Tan Y., Zhang T., Yang K., Zhu L. (2014). Superlow load of nanosized MnO on a porous carbon matrix from wood fibre with superior lithium-ion storage performance. J. Mater. Chem. A.

[39] Niyogi S., Bekyarova E., Itkis M.E., Zhang H., Shepperd K., Hicks J., Sprinkle M., Berger C., Lau C.N., deHeer W.A. (2010). Spectroscopy of covalently functionalized graphene. Nano Lett..

[40] Eckmann A., Felten A., Mishchenko A., Britnell L., Krupke R., Novoselov K.S., Casiraghi C. (2012). Probing the nature of defects in graphene by raman spectroscopy. Nano Lett..

[41] Zhao N., Zhi X., Wang L., Liu Y., Liang G. (2015). Effect of microstructure on low temperature electrochemical properties of LiFePO_4_/C cathode material. J. Alloys Compd..

[42] Yuan G., Bai J., Doan T.N.L., Chen P. (2015). Synthesis and electrochemical properties of LiFePO_4_/graphene composite as a novel cathode material for rechargeable hybrid aqueous battery. Mater. Lett..

[43] Xia Y., Zhang W., Huang H., Gan Y., Xiao Z., Qian L., Tao X. (2011). Biotemplating of phosphate hierarchical rechargeable LiFePO_4_/C spirulina microstructures. J. Mater. Chem..

[44] Paolella A., Bertoni G., Marras S., Dilena E., Colombo M., Prato M., Riedinger A., Povia M., Ansaldo A., Zaghib K. (2014). Etched Colloidal LiFePO_4_ Nanoplatelets toward High-Rate Capable Li-Ion Battery Electrodes. Nano Lett..

[45] Lee Y.C., Han D.-W., Park M., Jo M.R., Kang S.H., Lee J.K., Kang Y.-M. (2014). Tailored surface structure of LiFePO_4_/C nanofibers by phosphidation and their electrochemical superiority for lithium rechargeable batteries. ACS Appl. Mater. Interfaces.

[46] Wang G., Liu H., Liu J., Qiao S., Lu G.M., Munroe P., Ahn H. (2010). Mesoporous LiFePO_4_/C nanocomposite cathode materials for high power lithium ion batteries with superior performance. Adv. Mater..

[47] Zheng F., Yang C., Ji X., Hu D., Chen Y., Liu M. (2015). Surfactants assisted synthesis and electrochemical properties of nano-LiFePO_4_/C cathode materials for low temperature applications. J. Power Sources.

[48] Zhang L., Tang Y., Liu Z., Huang H., Fang Y., Huang F. (2015). Synthesis of Fe_2_P coated LiFePO_4_ nanorods with enhanced Li-storage Performance. J. Alloys Compd..

[49] Grosvenor A.P., Kobe B.A., Biesinger M.C., McIntyre N.S. (2004). Investigation of multiplet splitting of Fe 2p XPS spectra and bonding in iron compounds. Surf. Interface Anal..

[50] Zhang C., Liang Y., Yao L., Qiu Y. (2015). Effect of thermal treatment on the properties of electrospun LiFePO_4_-carbon nanofiber composite cathode materials for lithium-ion batteries. J. Alloys Compd..

[51] Miao C., Bai P., Jiang Q., Sun S., Wang X. (2014). A novel synthesis and characterization of LiFePO_4_ and LiFePO_4_/C as a cathode material for lithium-ion battery. J. Power Sources.

[52] Rao R.P., Reddy M.V., Adams S., Chowdari B.V.R. (2015). Preparation, temperature dependent structural, molecular dynamics simulations studies and electrochemical properties of LiFePO_4_. Mater. Res. Bull..

[53] Lin M., Chen Y., Chen B., Wu X., Kam K., Lu W., Chan H.L.W., Yuan J. (2014). Morphology-controlled synthesis of self-Assembled LiFePO_4_/C/RGO for high-performance Li-ion batteries. ACS Appl. Mater. Interfaces.

[54] Huang C., Kuo T.-R., Yougbaré S., Lin L.-Y. (2022). Design of LiFePO_4_ and porous carbon composites with excellent high-Rate charging performance for Lithium-Ion secondary battery. J. Colloid Interface Sci..

[55] Chen B., Liu M., Cao S., Hu H., Chen G., Guo X., Wang X. (2022). Direct regeneration and performance of spent LiFePO_4_ via a green efficient hydrothermal technique. J. Alloys Compd..

[56] Meng Y.-F., Liang H.-J., Zhao C.-D., Li W.-H., Gu Z.-Y., Yu M.-X., Zhao B., Hou X.-K., Wu X.-L. (2022). Concurrent recycling chemistry for cathode/anode in spent graphite/LiFePO_4_ batteries: Designing a unique cation/anion-co-work ab dual-ion battery. J. Energy Chem..

[57] Ding L., Leones R., Schmeida T., Nielsch K., Mikhailova D. (2022). Superior high-temperature rate performance of LiFePO_4_ cathode: The stabilizing effect of a multicomponent gel biopolymer binder. J. Power Sources.

[58] Li Y., Wang L., Zhang K., Liang F., Yao Y., Kong L. (2021). High performance of LiFePO_4_ with nitrogen and phosphorus dual-doped carbon layer for lithium-ion batteries. J. Alloys Compd..

[59] Zhuang Y., Zhang W., Bao Y., Guan M. (2022). Influence of the LiFePO_4_/C coating on the electrochemical performance of Nickel-rich cathode for lithium-ion batteries. J. Alloys Compd..

[60] Li P., Luo S.-H., Wang J., Wang Y., Wang Q., Zhang Y., Liu X., Gao D., Lv F., Mu W. (2022). Extraction and separation of Fe and Ti from extracted vanadium residue by enhanced ammonium sulfate leaching and synthesis of LiFePO_4_/C for lithium-ion batteries. Sep. Purif. Technol..

[61] Zhang T., Gong D., Lin S., Yu J. (2022). Effect of pH-dependent intermediate on the performance of LiFePO_4_/C cathode material. Chem. Eng. J..

[62] Fan M., Meng Q., Chang X., Gu C.-F., Meng X.-H., Yin Y.-X., Li H., Wan L.-J., Guo Y.-G. (2022). In Situ Electrochemical Regeneration of Degraded LiFePO4 Electrode with Functionalized Prelithiation Separator. Adv. Energy Mater..

[63] Liu B., Zhang Q., Li Y., Hao Y., Ali U., Li L., Zhang L., Wang C., Su Z. (2023). Realizing complete solid-solution reaction to achieve temperature independent LiFePO_4_ for high rate and low temperature Li-ion batteries. CCS Chem..

[64] Zhou S., Du J., Xiong X., Liu L., Wang J., Fu L., Ye J., Chen Y., Wu Y. (2022). Direct recovery of scrapped LiFePO_4_ by a green and low-cost electrochemical re-lithiation method. Green Chem..

[65] Wang X., Chen C., Wu S., Zheng H., Chen Y., Liu H., Wu Y., Duan H. (2022). High-rate and long-life Au nanorods/LiFePO_4_ composite cathode for lithium-ion batteries. Energy Technol..

[66] Zhu H., Xie Y., Zou S., Na B., Zeng R., Zhang S. (2022). Morphology control of free-standing LiFePO_4_-based composite membranes for advanced lithium-ion battery cathodes. Energy Technol..

[67] Lai A., Chu Y., Jiang J., Huang Y., Hu S., Pan Q., Zheng F., Wang J., Li J., Wang H. (2022). Self-restriction to form in-situ N, P co-doped carbon-coated LiFePO_4_ nanocomposites for high-performance lithium-ion batteries. Electrochim. Acta.

[68] Kovtyukhova N.I., Ollivier P.J., Martin B.R., Mallouk T.E., Chizhik S.A., Buzaneva E.V., Gorchinskiy A.D. (1999). Layer-by-layer assembly of ultrathin composite films from micron-sized graphite oxide sheets and polycations. Chem. Mater..

