# Supplementary material for: Reduced Graphene Oxide Coating LiFePO4 Composite Cathodes for Advanced Lithium-Ion Battery Applications
Source: Int J Mol Sci. 2023 Dec 16;24(24):17549. doi: 10.3390/ijms242417549 (PMC10743949; doi:10.3390/ijms242417549)
Supplement: Supplementary file 1 [file ijms-24-17549-s001.zip › ijms-2750420-supplementary.pdf]

*Electronic Supplementary Information*

# Reduced graphene oxide coating $LiFePO_4$ composite cathodes for advanced lithium-ion battery applications

Qingao Zhang [a], Yu Zhou [a], Yulong Tong [a], Yuting Chi [a], Ruhua Liu [a], Changkai Dai [a], Zhanqing Li [a], Zhenli Cui [a], Yaohua Liang [b*], and Yanli Tan [a*]

[a] School of Chemical Science and Engineering, Qingdao University, Qingdao 266071, PR China

[b] Department of Agricultural and Biosystems Engineering, South Dakota State University, Brookings, SD 57007, USA

*** Corresponding author:**

*E-mail address: yanlitantan@qdu.edu.cn* (Y. L. Tan), yaohua.liang@sdstate.edu (Y. H. Liang).

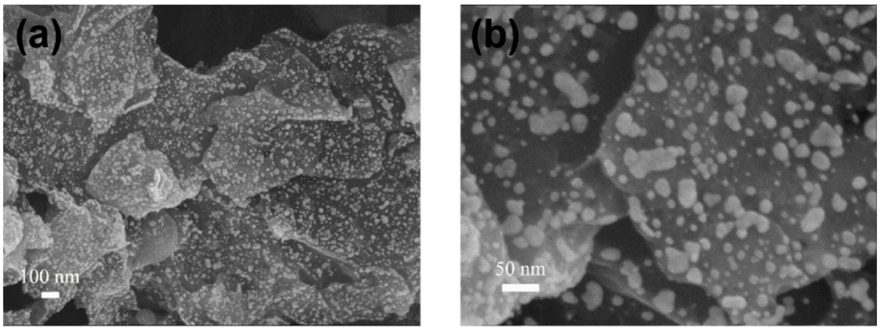


**Figure S1.** SEM images of LFP/rGO-300 composite (a, b).

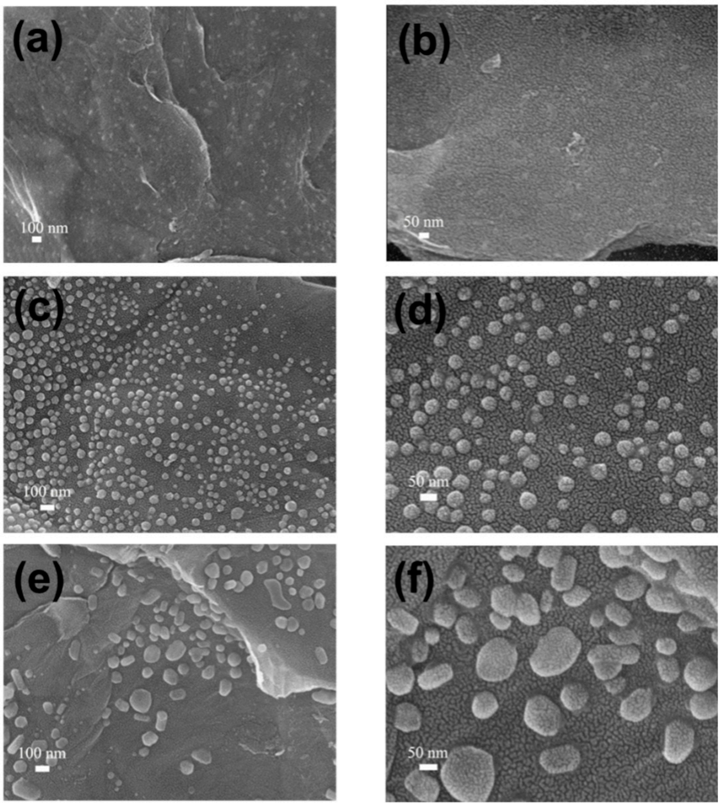


**Figure S2.** SEM images of LFP/rGO-200 (a, b), LFP/rGO-400 (c, d) and LFP/rGO-500 (e, f).

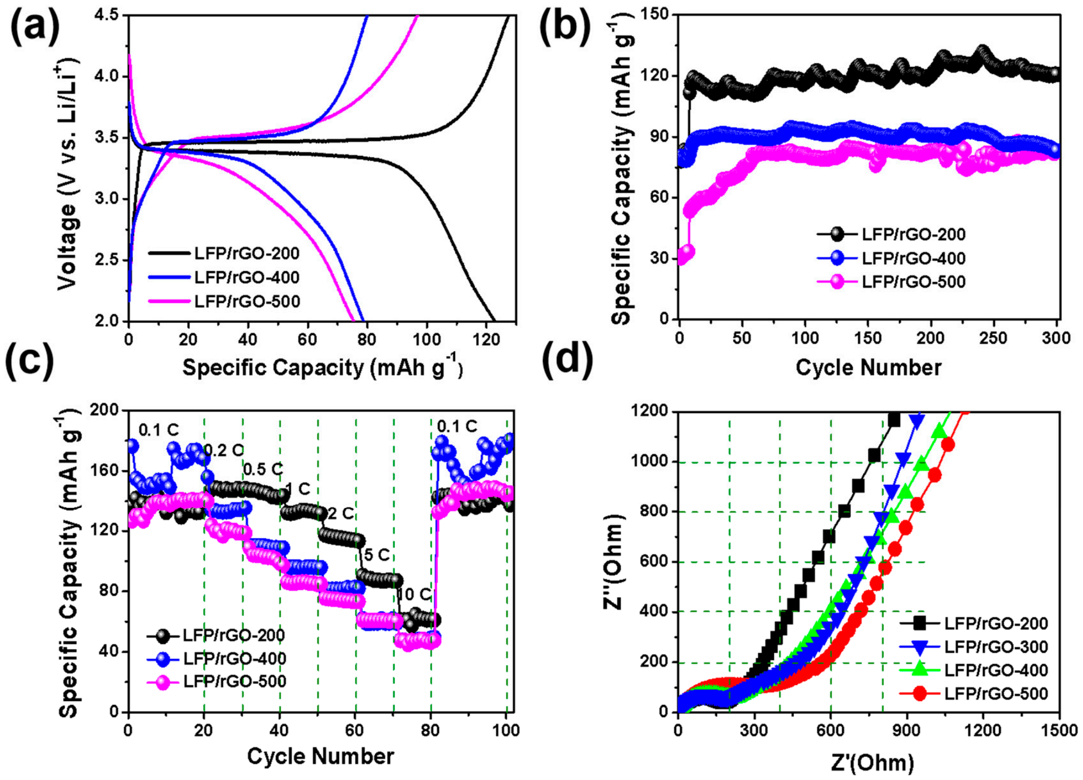


**Figure S3.** Charge-discharge curves at current density of 1 C (a), Cycling performance at current density of 1 C (b) Rate-performance (c) and Nyquist plots (d) of LFP/rGO-200, LFP/rGO-400 and LFP/rGO-500 composites.

Table S1. Comparison of electrochemical Li-ion storage of LFP/rGO-300 composite (current study) with other LFP-based literature reports (1C =170 mA $g^{-1}$ ).

| Sample | Capacity (mA h $g^{-1}$ ) at low rate (C) | Capacity (mA h $g^{-1}$) at high rate (C) | Capacity (mAh $g^{-1}$) at x rate (C) after multiple numbers | Cycle number | Capacity retention | Ref. |
|---|---|---|---|---|---|---|
| LFP@OFC | 169.9(0.1C) | 85.6 (16.2C) | 160.9(1C) | 500 | - | 8 |
| $Zn(OAc)_2$·DEA -based LFP | 169 (0.2C) | 86(20C) | 80(5C) | 1500 | 56% | 10 |
| $LiFePO_4$/C | 151.6(0.1C) | 121.9 (10C) | 120.5(10C) | 100 | 98.83% | 12 |
| LFP@CT | 157.81(0.1C) | 121.26(5C) | - | 100 | 99.88% | 13 |
| N-C@LFP | 136.65(0.2C) | 114.55(1C) | 113.51(1C) | 200 | 99.1% | 17 |

| | | | | | | |
|---|---|---|---|---|---|---|
| L-CGC5-2.5-2.5 | 163.7(0.2C) | 69.9 (20C) | 93.4(5C) | 300 | 81.3% | 19 |
| LFP/C-SP5 | 165.6(0.1C) | 59.8 (10C) | 54(3C) | 500 | 36% | 54 |
| R-LFP-700 | 165.9(0.1C) | 114.96 (5C) | 144.6(1C) | 200 | 99.1% | 55 |
| LFP/graphite | 117.4(0.15C) | 76.2 (3C) | 74.3 (0.59C) | 1000 | 78% | 56 |
| LFP/PGB | - | 113 (10C) | 57 (10C) | 1000 | 50.4% | 57 |
| LFP@NPC | 150.2(1C) | 119.7 (10C) | 140.1(2C) | 300 | 99.5% | 58 |
| NCM811@ 1LFP/C | 188.8(0.1C) | 143.5 (2C) | 109.1(2C) | 300 | 76% | 59 |
| $LiFePO_4$/C | 142.2(0.1C) | 97 (5C) | 132.7(0.1C) | 50 | 93.36% | 60 |
| LFP-1.5 | 128.36(1C) | 93.14(10C) | 132.0 (1C) | 100 | 106.31% | 61 |
| D-LFP | 159(0.1C) | 139.4(2C) | 146.7(0.1C) | 292 | 90.7% | 62 |
| PNCsLFP | 166.1(0.2C) | 146.1(10C） | 157.6(1C) | 500 | 98.5% | 63 |
| R-LFP-OP | 137.2(0.2C) | 108.5(5C) | 134(1C) | 300 | 85.5% | 64 |
| LFP/Au NRs | 151.5(1C) | 91.9(5C) | 121.3(1C) | 200 | 80% | 65 |
| $LiFePO_4$-KB-BC | 140(1C) | 116(5C) | 137.2(1C) | 300 | 98% | 66 |
| LFP/N, P-C | 155(0.1C) | 123 (10C) | 127(5C) | 1000 | 94.5% | 67 |
| LFP/rGO-300 | 168(0.1C) | 88(10C) | 80(10C) | 1000 | 91% | Our work |
